# 6-methylmercaptopurine riboside, a thiopurine nucleoside with antiviral activity against canine distemper virus in vitro

**DOI:** 10.1186/s12985-017-0785-6

**Published:** 2017-06-26

**Authors:** Otávio Valério de Carvalho, Daniele Mendes Félix, Claudia de Camargo Tozato, Juliana Lopes Rangel Fietto, Márcia Rogéria de Almeida, Gustavo Costa Bressan, Lindomar José Pena, Abelardo Silva-Júnior

**Affiliations:** 1 Laboratory of Animal Virology, Department of Veterinary, Universidade Federal of Viçosa, Av. Peter Henry Rolfs, s/n, Viçosa, MG 36570-000 Brazil; 2 Laboratory of Virology and Experimental Therapy, Oswaldo Cruz Foundation (FIOCRUZ), Aggeu Magalhães Research Center, Av. Moraes Rego, s/n, Campus UFPE, Cidade Universitária, Recife, PE 50670-420 Brazil; 3 Laboratory of Animal and Human Virology, Department of Microbiology and Immunology, Biosciences Institute, Paulista State University, Botucatu, SP 18618-970 Brazil; 4 Department of Biochemistry and Molecular Biology, Federal University of Viçosa, Av. Peter Henry Rolfs, s/n, Viçosa, MG 36570-000 Brazil

**Keywords:** Canine distemper, Antiviral, Azathioprine, Thiopurine, Nucleoside analogue

## Abstract

**Background:**

Canine distemper (CD) is a widespread infectious disease that can severely impact a variety of species in the order Carnivora, as well as non-carnivore species such as non-human primates. Despite large-scale vaccination campaigns, several fatal outbreaks have been reported in wild and domestic carnivore populations. This, in association with expansion of the disease host range and the development of vaccine-escape strains, has contributed to an increased demand for therapeutic strategies synergizing with vaccine programs for effectively controlling canine distemper. 6-methylmercaptopurine riboside (6MMPr) is a modified thiopurine nucleoside with known antiviral properties against certain RNA viruses.

**Methods:**

We tested the inhibitory effects of 6MMPr against a wild-type CDV strain infection in cell culture. We measured infectious particle production and viral RNA levels in treated and untreated CDV-infected cells. Ribavirin (RIB) was used as a positive control.

**Results:**

Here, we report for the first time the antiviral effects of 6MMPr against canine distemper virus (CDV) in vitro. 6MMPr was able to reduce viral RNA levels and to inhibit the production of infectious CDV particles. The therapeutic selectivity of 6MMPr was approximately six times higher than that of ribavirin.

**Conclusion:**

Our results indicate that 6MMPr has high anti-CDV potential and warrants further testing against other paramyxoviruses, as well as clinical testing of the compound against CDV.

## Background

Canine distemper (CD) is a multisystemic infectious disease caused by canine distemper virus (CDV), which affects a broad range of domestic and wild carnivores worldwide, resulting in high mortality rates. Despite its huge relevance for dog populations, CD also impacts endangered and threatened animals [1] and, more recently, has been reported in non-carnivorous species such as non-human primates [2]. Canine distemper infection is characterized by respiratory and gastrointestinal disorders, often accompanied by immunosuppression and neurological complications in most infected hosts [3]. A close relative of the measles virus (MV), CDV is a highly contagious, enveloped single-stranded negative RNA virus belonging to genus *Morbillivirus* within the *Paramyxoviridae* family of the Mononegavirales order. The CDV genome encodes six structural proteins: hemagglutinin (H), fusion (F), envelope-associated matrix (M), phospho- (P), large polymerase (L) and nucleoprotein (N) [4].

Vaccination is an effective tool to prevent infectious diseases [5]. The extensive vaccination of domestic dogs has greatly reduced the incidence of canine distemper. Nevertheless, there is a growing concern about CDV genetic variability regarding the cell binding site of the H protein. Mutations in the H gene might be associated with antigenic divergence between field strains, current vaccine viruses [6], and host-switching events [7]. Antigenic drift and the absence of a specific treatment may hamper effective control and eradication of CDV [8, 9].

Several synthetic compounds have been described as potent inhibitors of paramyxoviruses [10, 11]. Ribavirin (RIB, 1-β-D-ribofuranosyl-1,2,4-triazole-3-carboxamide) is a purine nucleoside analogue that displays antiviral effects against DNA and RNA viruses. RIB is incorporated into growing viral genomes, resulting in lethal mutagenesis [12]. Previous studies showed that RIB strongly decreases CDV replication in vitro [13–16].

Azathioprine (AZA) is a thiopurine prodrug used widely as an immunosuppressant. AZA is converted to 6-mercaptopurine (6MP), which is further transformed to several active metabolites [17]. In a competitive enzymatic pathway, thiopurine methyltransferase (TPMT) catalyzes the S-methylation of 6MP to produce 6-methylmercaptopurine riboside (6MMPr). 6MMPr is known to inhibit purine synthesis, and its antiviral effects have been demonstrated against flaviviruses such as hepatitis C virus (HCV) RNA replicon, bovine viral diarrhea virus (BVDV) [18, 19], yellow fever virus (YFV), dengue virus-2 (DENV-2), and West Nile virus (WNV) [20]. Among the thiopurine metabolites, 6MMPr showed the greatest antiviral potential [19]. Here, we show for the first time that CDV production can be markedly inhibited by 6MMPr in cell culture.

## Methods

### Cell culture and virus

VerodogSLAM cells were grown in Dulbecco’s modified Eagle’s medium (DMEM High Glucose, D7777, Sigma-Aldrich, Saint Louis, USA) supplemented with 10% fetal bovine serum (FBS, Gibco), 1% penicillin/streptomycin and 1 mM L-glutamine (Gibco) at 37 °C in a humidified incubator with 5% CO_2_. Zeocin (Invitrogen) was added at 1 mg/mL for stable maintenance of SLAM (signaling lymphocytic activation molecule) tag expression. A CDV field strain (CDV/LDM-BTU-2, GenBank accession number KX434626) isolated from canine clinical specimens was used in this study. The wild-type CDV was propagated on VerodogSLAM cells and virus stocks were prepared by collecting the infected cells plus supernatant (SUP) when the cytopathic effect (CPE) was ~80%; samples were stored in aliquots at −80 °C. Virus titers were determined by the TCID_50_ (50% tissue culture infective dose) method of Reed and Muench [21] and expressed as log_10_ TCID_50_/mL.

### 6-Methylmercaptopurine riboside

**Fig. 1:**
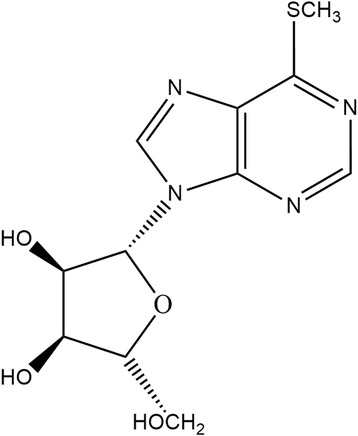
Chemical structure of 6-methylmercaptopurine riboside (6MMPr)

6-methylmercaptopurine riboside (6MMPr) (Fig. 1) and 1-β-D-ribofuranosyl-1,2,4-triazole-3-carboxamide (ribavirin) were purchased from Sigma-Aldrich (Saint Louis, USA). Ribavirin (RIB) was used as the positive control. Stock solutions of the compounds were prepared in Milli-Q H_2_O and sterilized by filtering through a Millipore 0.22 μM filter. All stock solutions were stored at −20 °C and the working solutions were prepared immediately before the start of each experiment.

### Cell viability assay

The cell toxicity of 6MMPr and RIB was tested on growing cells via in situ mitochondrial reduction of a tetrazolium dye, 3-(4,5-dimethylthiazol-2-yl)-2,5-diphenyltetrazolium bromide (MTT) (Sigma, St. Louis, USA). Briefly, VerodogSLAM cells (1 × 10^4^ cells/well) were seeded on 96-well microplates for 24 h. The medium was replaced with 200 μL fresh DMEM containing different concentrations of the compounds. After 72 h of incubation at 37 °C, the culture medium was removed and replenished with 50 μL of MTT working solution (1 mg/mL) to each well and the microplate was incubated for 4 h. MTT formazan crystals were solubilized by adding DMSO (dimethyl sulfoxide) and the optical densities were determined spectrophotometrically with an absorbance microplate reader (BioTek, ELX800, Winooski, Vermont, USA) at 540 nm. Cell viability was calculated by subtracting the optical density fraction of treated cells from the untreated cells. The cytotoxic concentration for 50% of the cell culture (CC_50_) was expressed as the compound concentration required to reduce the absorbance of treated cells by 50% in comparison to control cells. CC_20_ was defined as the limit point for treatment with the antiviral molecules [22]. At least two independent experiments were performed with eight replicates for each concentration level.

### Virus infection and 6MMPr treatment

VerodogSLAM cells were grown a day prior in 24-well tissue culture plates at a density of 10^5^ cells/well in DMEM supplemented with 10% FBS. Cells were first infected with CDV/LDM-BTU-2 at a multiplicity of infection (MOI) of 0.1 and incubated for 2 h (37 °C, 5% CO_2_). After virus internalization, viral inoculum was removed, cells were washed twice with DMEM and replaced with fresh medium containing several two-fold dilutions of the compounds tested (338, 169, 84.5, 42 μM of 6MMPr and 81.5, 40.8, 20.4, 10.2 μM of RIB). Infected non-treated and mock infected controls were set up in parallel. At 72 h post-infection (PI), 24-well plates were freeze/thawed once, and cell lysates plus SUP were harvested and stored at −80 °C until use for virus titration and real-time quantitative RT-PCR analysis. Virus titers are expressed as log_10_ TCID_50_/mL according to the method of Reed and Muench [21]. All antiviral assays were carried out in triplicate, and the results are shown as the mean values ± standard errors obtained from at least two individual experiments.

### Plaque-reduction assay for assessment of antiviral activity

Antiviral efficacy of 6MMPr was also evaluated by measuring the reduction in the number of CDV infectious plaques. Confluent monolayers of VerodogSLAM cells by 2 × 10^5^ cells/well were seeded in 24-well plates and incubated for 24 h. Cell lysate + SUP from the various antiviral drug treatments, including RIB, were added to cells and, after 2 h of incubation, the inocula were removed and the cells were overlaid with DMEM supplemented with 2.5% carboxymethyl cellulose (CMC) and 2% FBS. The plates were incubated for 72 h at 37 °C in a 5% CO_2_ incubator. Monolayers were fixed using 10% formalin in phosphate-buffered saline and then stained with crystal violet solution; plaque numbers were then quantified. The percentage of plaque reduction (PR%) in comparison to untreated infected cells was calculated using the following formula: PR (%) = (C - T) × 100/C, where C is the mean number of plaques from triplicate untreated control wells and T is the mean number of plaques from triplicate treated wells. The 50% inhibitory concentration (EC_50_) was defined as the compound concentration required to reduce the CDV plaque count by 50% of the virus control. The selectivity index (SI) was obtained by calculating the ratio of the CC_50_ and the EC_50_ values.

### Assessment of antiviral effect of 6MMPr at different time points post-infection

Confluent 24 h–plated VerodogSLAM cells seeded into 24-well tissue culture plates were infected with CDV/LDM-BTU-2 at an MOI of 0.1. After an incubation time of 2 h at 37 °C in a 5% CO_2_ atmosphere, the viral inoculum was removed and replaced by fresh medium containing 338 μM of 6MMPr. At different time points PI (24, 48 and 72 h), cells plus SUP were collected for virus titer estimates from the TCID_50_, and viral RNA was extracted and quantified by qRT-PCR.

### Addition of 6MMPr at different time points post-infection

VerodogSLAM cells were first infected with CDV/LDM-BTU-2 at an MOI of 0.1. After 2 h of incubation at 37 °C and 5% CO_2_, the viral inoculum was removed and replaced with culture medium containing 2% FBS. At several time points PI (2, 12 and 24 h), 6MMPr 338 μM was added to the infected cells. At each time point, virus and cell controls were included in the assay. At 72 h post viral infection, 24-well plates were freeze/thawed once, and cell lysates plus SUP were harvested and stored at −80 °C until use for TCID_50_ virus titration and qRT-PCR analysis.

### Quantification of viral RNA by real-time RT-PCR

Total RNA was extracted from cell lysate plus SUP (500 μL) using Trizol reagent (Invitrogen) according the manufacturer’s instructions. The concentration and quality of RNA was checked using a NanoDrop 2000 spectrophotometer (Thermo Fisher Scientific). For each real-time assay, 100 ng of RNA template was analyzed by qRT-PCR using GoTaq 1-Step RT-qPCR System kit (Promega). CDV-specific primers for nucleoprotein gene (N) CDV-F (AGTTAGTTTCATCTTAACTATCAAATT) and CDV-R (TTAACTCTCCAGAAAACTCATGC) had been previously designed [23]. Primer sets were synthesized by Integrated DNA Technologies (IDT). RNA measurement by SYBR green incorporation was carried out using an Applied Biosystems 7500 Real-Time PCR System (Applied Biosystems) with the following thermal cycling profile: reverse transcription at 50 °C for 30 min, activation of Taq polymerase at 95 °C for 10 min and 40 cycles consisting of denaturation at 95 °C for 10 s, annealing at 60 °C for 30 s and polymerization at 72 °C for 30 s. At the end of amplification, a melt curve was performed from 70 °C to 95 °C and fluorescence data were collected every 0.3 °C during melting. Real-time RT-PCR data were analyzed with Applied Biosystems 7500 Software v2.0.6 (Applied Biosystems). CDV RNA levels in treated and control cells were determined by absolute quantification with the standard curve method. Briefly, a 287-bp fragment resulting from conventional PCR with primers P1 (ACAGGATTGCTGAGGACCTAT) and P2 (CAAGATAACCATGTACGGTGC) described by Frisk et al. [24] was cloned into the pGEM-T Easy vector (Promega). The pGEM-inserted fragment contained the amplicon sequence used for the real-time qRT-PCR assay. After confirming and linearizing the pGEM-T Easy construct with the *Spel* restriction enzyme (Promega), the linearized plasmid was used as a template for in vitro transcription with the MEGAshortscript T7 Transcription Kit (Ambion), according to the manufacturer’s instructions. Turbo DNase-treated transcripts were ethanol-precipitated and resuspended in RNAse-free water. Synthetic RNAs were quantified using a Qubit 2.0 Fluorometer (Thermo Fischer Scientific) and the copy number was determined using the following formula: (X g/μL DNA/[transcript length in base pairs × 340]) × 6.022 × 10^23^ = Y ssRNA molecules/μL. Standard curves were constructed with five points in triplicate from serial 10-fold dilutions of transcripts. RNA samples were tested in duplicate and the inhibition of CDV replication was expressed as RNA copy number.

### Statistical analysis

Statistical analysis was performed to assess the differences in viral yield of infected cells in contact with 6MMPr for different doses and time intervals. Data were submitted to two-way ANOVA (analysis of variance) using GraphPad Prism Software version 5.01 for Windows (GraphPad Software, La Jolla, California, USA). The Tukey test was carried out to perform pairwise comparisons among means. Values of CC_50_ and EC_50_ were calculated from a linear regression equation. All the graphs show the mean ± standard error from three independent experiments. A *p*-value <0.05 was considered statistically significant.

## Results

### Cytotoxicity and antiviral effects of 6MMPr and RIB against CDV

Cytotoxicity data were analyzed to determine the non-toxic concentrations of 6MMPr and RIB to VerodogSLAM cells. 6MMPr was 3.3 times less toxic in comparison to RIB. The average CC_50_ values of 6MMPr and RIB were 1409 μM and 424 μM, respectively (Table 1). The maximum non-toxic concentrations (MNTC) employed in the antiviral assays, previously defined as CC_20_, were 338 μM for 6MMPr and 81.5 μM for RIB, and none of them induced any visible cell morphological changes.

**Table 1:** Cytotoxicity, antiviral activity, and selectivity indexes of 6MMPr and RIB

Compounds	Cytotoxicity CC_50_ (μM)^a^	Antiviral activity				
		EC_50_ (μM)^a^	SI^b^	Log_10_ reduction value^d^		
				CC_20_ (μM)^c^	qRT-PCR	TCID_50_
6MMPr	1409	28.7	49.1	338	1.3	>2.2
RIB	424	52	8.2	81.5	0.4	0.6

^a^CC_50_ (50% cytotoxic concentration) and EC_50_ (50% cytotoxic concentration) mean values

^b^Selectivity index (CC_50_/EC_50_)

^c^CC_20_ (20% cytotoxic concentration): maximum non-toxic concentration employed in the antiviral assays

^d^Log_10_ reduction was calculated by subtracting the log_10_ means of the CDV infectivity in the presence of CC_20_ compounds from the log_10_ means of the CDV infectivity in the untreated cells

**Fig. 2:**
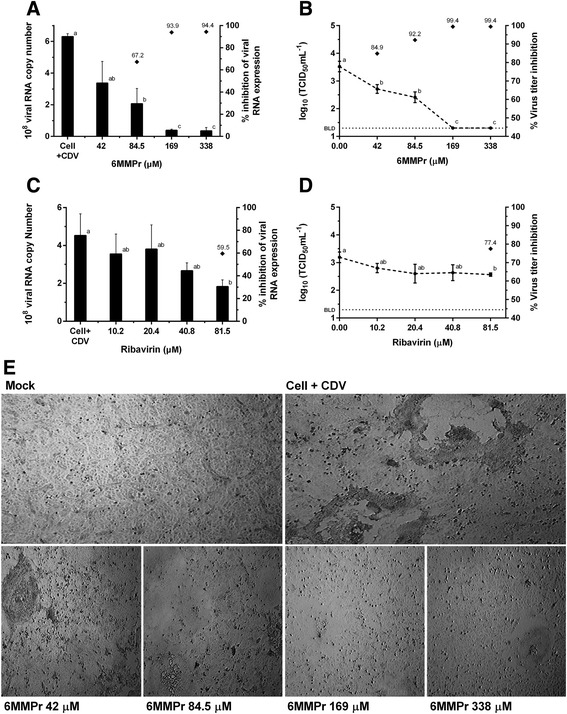
Antiviral activity assay of 6MMPr and RIB. CDV production was measured in the presence of several two-fold dilutions of the tested compounds. Real-time qRT-PCR and TCID_50_ infectivity titration of 6MMPr (**a**, **b**) and RIB (**c**, **d**). *Right vertical axis* presents the percentage of virus inhibition highlighted on the markers (♦). *Error bars* represent standard deviations. Values are the mean ± standard error obtained from three independent experiments. Values followed by the same lowercase letter do not differ by Tukey’s test (*p* < 0.01). BLD, below limit of detection for TCID_50_ method (20 TCID_50_/mL). **e** VerodogSLAM cells were mock-infected or CDV-infected (MOI 0.1) and treated with 6MMPr at different concentrations (100× total magnification)

Analysis of the antiviral activity assays demonstrated a 6MMPr dose-dependent inhibition of CDV replication (Fig. 2). We observed a clear reduction in CPE levels in the infected cells treated with 6MMPr. At the highest concentrations (169 and 338 μM), 6MMPr reduced CDV RNA copy number by 94%, and there were no virus particles detected by TCID_50_ assay. RIB showed lower antiviral effects with significant inhibition of the virus transcript level by 60% and a virus titer reduction of 77%, which was displayed only at the highest concentration (81.5 μM). While RIB activity decreased approximately 0.5 log_10_ of viral growth and RNA synthesis, 6MMPr reduced up to 1.3 log_10_ of virus RNA and over 2.2 log_10_ of infectious titers (Table 1).

**Fig. 3:**
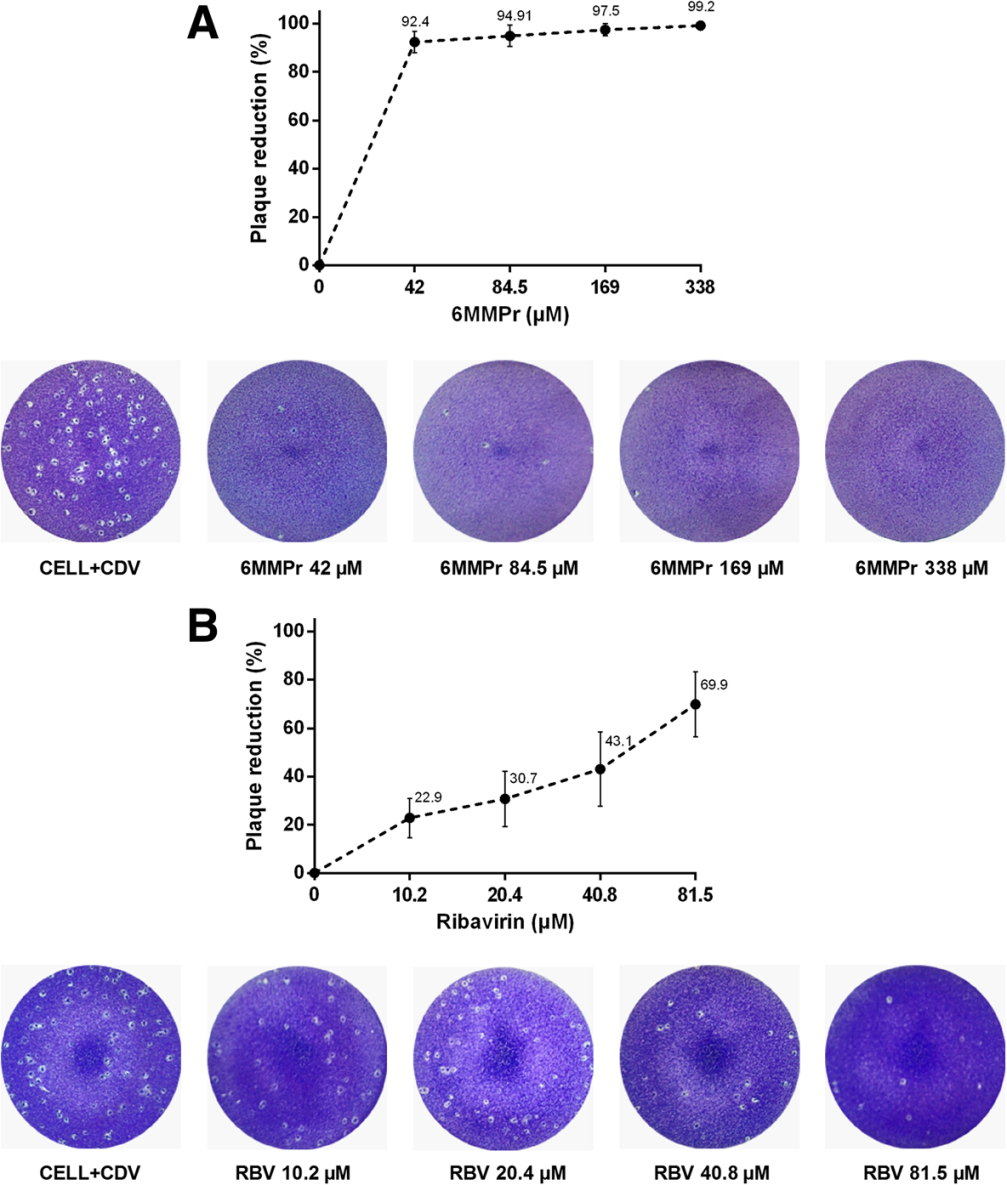
CDV plaque-reduction assay with 6MMPr and RIB treatments. VerodogSLAM cells were inoculated with samples of antiviral activity assays. Plaque reduction values of 6MMPr (**a**) and RIB (**b**) assays represent the average ± SD from three independent experiments compared with untreated infected cells. Plaques were visualized by crystal violet staining

The plaque-reduction assay (PRA) was used to confirm compound activity and to determine the 50% inhibitory concentration (EC_50_). PRA analysis showed strong agreement with antiviral activity assays. 6MMPr and RIB exhibited EC_50_ values of 28.7 and 52 μM, respectively. As shown in Table 1, the SI value of 6MMPr was 49.1, which is approximately six times higher than the SI value of 8.2 for RIB. 6MMPr displayed the greatest effects regarding inhibition of CDV-induced plaques, with a maximum reduction of 99%. Indeed, it caused a clear decrease in plaque number and size (Fig. 3).

### Time-dependent inhibition

**Fig. 4:**
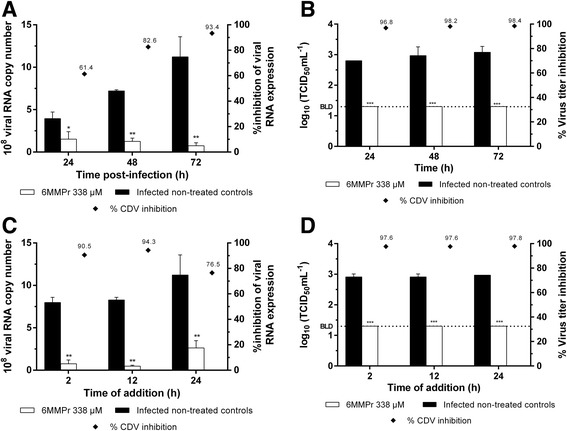
Time-dependent inhibition (**a**, **b**) and time-of-drug addition approach (**c**, **d**) for 6MMPr. CDV-infected cells were treated with 6MMPr (338 μM) and the viral load was measured by qRT-PCR (**a**) and the TCID_50_ method (**b**) at different time points (24, 48 and 72 h) PI. With time-based addition, CDV-infected cells were treated with 6MMPr (338 μM) at 2, 12 and 24 h PI, and inhibition efficiency was quantified with qRT-PCR (C) and TCID_50_ titration (D). The *right vertical axis* presents the percentage of virus inhibition highlighted by the markers (♦). The *error bars* represent standard deviation. Values are the mean ± standard error obtained from three independent experiments (* *p* < 0.05, ** *p* < 0.01, *** *p* < 0.001). BLD, below limit of detection for the TCID_50_ method (20 TCID_50_/mL)

We evaluated the reduction of CDV RNA levels and virus infectious titers in the presence of 6MMPr 338 μM at different times PI (Fig. 4a, b). TCID_50_ virus titration and absolute quantitative real-time RT-PCR methods were used to measure the virus yield from cells plus SUP collected at the 24, 48 and 72 h time points. We observed that 6MMPr was able to reduce RNA synthesis at all time points. Virus titration showed that 6MMPr could completely inhibit viral growth at all measured time points.

### Antiviral effect of addition of 6MMPr at different time points post-infection

To investigate the 6MMPr antiviral activity at different time points post-infection, a time-based approach was carried out by adding 6MMPr 338 μM at 2, 12 and 24 h after virus infection. The CDV RNA level measured at 2 and 12 h showed no difference for 6MMPr activity. The observed viral RNA reduction at 2 and 12 h ranged from 90.5 to 94.3%, and was greater than that at 24 h (76.5%). As shown in Fig. 4c and d, although the CDV RNA level varied between time points, 6MMPr completely inhibited viral infectivity at each time point.

## Discussion

Canine distemper virus causes a devastating multisystemic disease in domestic and wild carnivores, with high morbidity and mortality rates. Furthermore, there are growing concerns about the impact of CDV infection because of its recurrent outbreaks, conservation threat to wildlife, economic impact, the emergence of vaccine-escaping strains, host-switching events, and potential risk for humans [7, 8, 25]. Therefore, the discovery of antiviral drugs able to effectively control CDV is an urgent need. We report here novel data showing that 6-methylmercaptopurine riboside induces highly efficient inhibition of CDV replication. 6MMPr is a modified thiopurine nucleoside derived from the catalyzed biotransformation of the prodrug azathioprine, and has already been reported to inhibit the replication of diverse viruses. Among the AZA metabolites, 6MMPr showed the highest antiviral efficacy against BVDV. Treatment with 6MMPr had also shown inhibitory effects against HCV and YFV replicon, but HCV had higher susceptibility to inhibition by 6MMPr than YFV replicon. Similar results have been found for DENV-2 and WNV, both were inhibited by the drug, but DENV-2 was more susceptible to 6MMPr than WNV. Replications kinetics and conformational changes of viral polymerase could be possible explanations for such efficacy differences [18–20].

In this study, 6MMPr showed inhibition effects against a wild-type virulent CDV strain. At the highest concentrations (169 and 338 μM), 6MMPr was able to reduce the production of infectious particles to undetectable levels. The antiviral properties of 6MMPr are dependent upon virus specificity and presumably acts by blocking viral RNA replication [19]. Taking ribavirin as a reference compound for anti-CDV activity, 6MMPr achieved therapeutic selectivity approximately six times higher in comparison to RIB. Likewise, given the proposed mechanism of action of RIB, active AZA metabolites could be incorporated into the viral RNA after being phosphorylated, resulting in increased mutations [26], disruption to the RNA structural conformation, or RNA chain-terminating events [27]. Besides inhibition of the purine de novo biosynthetic pathway and incorporation into nucleic acids, 6MMPr probably also has direct inhibitory effects on viral polymerase [28] and may be related to inhibiting the initiation step of the virus replication process [19]. These mechanisms, alone or in combination, could explain the antiviral activity of 6MMPr on CDV replication, but the precise target of action remains elusive and should be explored in future studies.

Although the details of CDV replication are not thoroughly understood, the eclipse phase was found to be approximately 16 h and the complete replication cycle takes place approximately 24 h after the virus penetrates the cellular membrane [29]. In order to investigate the possible CDV replication targets of 6MMPr and how long the addition of the compound could be postponed before decreasing its antiviral efficiency, we performed a time-based 6MMPr treatment at 2, 12 and 24 h PI. The compound was active at all measured time points, but there was lower viral RNA inhibition at 24 h, which was not correlated with an increased infectious virus load at that time. These findings show that 6MMPr exhibited inhibitory activity even when added at later time points post-infection. Further investigations are needed to identify the stage of the viral replication cycle that is likely being affected by 6MMPr.

Initially, negative-stranded RNA viruses direct the RNA synthesis machinery to mRNA production. Replication processes are then initiated [30]. Both transcription and genome replication events of paramyxoviruses are mediated by the single encoded RNA polymerase [31]. In this way, the viral RNA-dependent RNA polymerase (RdRp) complex remains available during different phases of the paramyxovirus replication cycle and becomes an attractive target for therapeutic intervention [11]. Since 6MMPr has been found to inhibit BVDV RdRp [28], perhaps the CDV L polymerase could also be a potential target site for 6MMPr therapy; this should be checked in future mechanism-of-action studies.

While we found that antiviral activity of RIB achieved a maximum reduction in CDV infectivity of 0.6 log_10_, previous studies reported that RIB was able to decrease between 1 and 5 log_10_ of CDV infectious titer in Vero cells. However, we found an EC_50_ of 12.7 μg/mL (52 μM) for RIB, which is close to the average EC_50_ values of RIB previously exhibited for CDV inhibition [13–16]. There was no consensus in these studies regarding the toxicity of RIB in Vero cells, since CC_50_ mean values ranged between 27.2 μg/mL [4] to over 3907 μg/mL (>16 mM) [14]. Hence, the RIB selectivity indexes also presented variable values ranging from 1.07 [15] to 42.4 [16]. It is not possible to make valid comparisons regarding RIB activity against CDV because we employed a different cell type; unlike all previous studies, we used a wild-type CDV instead of a vaccine strain in the antiviral assays with RIB.

## Conclusion

Together, our results show that 6MMPr is highly effective at inhibiting CDV. The compound exhibited strong inhibitory potential even when added subsequent to viral challenge. More studies are needed to gain further insight into the precise mechanisms of 6MMPr anti-CDV activity. Thus, 6MMPr represents a promising candidate for clinical applications against CDV infection.

## Abbreviations

6MMPr: 6-methylmercaptopurine riboside
6MP: 6-mercaptopurine
AZA: Azathioprine
BVDV: Bovine viral diarrhea virus
CC_50_: Cytotoxic concentration for 50% cell culture
CD: Canine distemper
CDV: Canine distemper virus
CMC: Carboxymethyl cellulose
CPE: Cytopathic effect
DENV: Dengue virus
DMEM: Dulbecco’s modified Eagle’s medium
DMSO: Dimethyl sulfoxide
EC_50_: 50% inhibitory concentration
F: Fusion protein
FBS: Fetal bovine serum
H: Hemagglutinin
HCV: Hepatitis C virus
L: Large polymerase
M: Envelope-associated matrix protein
MNTC: Maximum nontoxic concentration
MTT: 3-(4,5-dimethylthiazol-2-yl)-2,5-diphenyltetrazolium bromide
N: Nucleoprotein
P: Phosphoprotein
RIB: Ribavirin, 1-β-D-ribofuranosyl-1,2,4-triazole-3-carboxamide
SI: Selectivity index
SLAM: Signaling lymphocytic activation molecule
TCID_50_: 50% tissue culture infective dose
TPMT: Thiopurine methyltransferase
WNV: West Nile virus
YFV: Yellow fever virus
